# Understanding test accuracy research: a test consequence graphic

**DOI:** 10.1186/s41512-017-0023-0

**Published:** 2018-02-01

**Authors:** Penny Whiting, Clare Davenport

**Affiliations:** 1 NIHR CHLARC West, University Hospitals Bristol NHS Foundation Trust, Bristol, UK; 2 Bristol Medical School, University of Bristol, Bristol, UK; 3 Institute of Applied Health Research, University of Birmingham, Birmingham, UK

**Keywords:** Diagnosis, Natural frequencies, Graphical display

## Abstract

**Background:**

Presenting results of diagnostic test accuracy research so that it is accessible to users is challenging. Commonly used accuracy measures (e.g. sensitivity and specificity) are poorly understood by health professionals and the public. Evidence suggests that presenting probabilities as natural frequencies rather than percentages facilitates understanding. We present a test consequence graphic to display results based on natural frequencies and test consequences.

**Methods:**

The graphic was developed as part of a project to develop guidance for writing plain language summaries for Cochrane diagnostic test accuracy (DTA) reviews. Using a mixed methods approach (focus groups, user testing, web-based surveys, public engagement and piloting), the graphic emerged as a clear preference out of a range of methods for presenting probabilistic information (text only, numbers embedded in text, annotated graphic) across patient representatives, media representatives and health professionals. The structure of the graphic was refined during the research process.

**Results:**

The test consequence graphic displays the results of diagnostic test accuracy study or review as natural frequencies based on a hypothetical cohort of 1000 patients receiving the test.

**Conclusions:**

The test consequence graphic provides a tool to help researchers communicate the results of diagnostic research in a simple, easy to access format and encourage meaningful application of research findings to practice. Key to this is linking estimates of test accuracy to potential downstream consequences of testing.

**Electronic supplementary material:**

The online version of this article (10.1186/s41512-017-0023-0) contains supplementary material, which is available to authorized users.

## Background

Explaining the results of test accuracy research in plain language is challenging. Outcome measures such as sensitivity and specificity are less familiar and more poorly understood than outcome measures used in intervention studies (e.g. relative risk) [1]. The two-dimensional nature of the measure of a test’s accuracy (the accuracy of a positive test result separate to the accuracy of a negative test result) introduces further complexity.

Research suggests that natural frequencies may facilitate understanding of probabilistic information and better estimation of the post-test probability of disease [1, 2]. In addition, simultaneous presentation of probabilistic information using textual explanation and numeric and graphic formats are preferred over the use of any of these presentation formats alone [3–6]. These findings have important implications for how the results of test accuracy research should be presented.

In this article, we introduce a graphic (the “test consequence graphic”) to present the results of test accuracy research, both primary studies and systematic reviews. The graphic is based on a natural frequency representation of the results that would be expected if the test under evaluation were applied to a hypothetical cohort of patients suspected of having the disease (target condition) for which the test is being used. It also incorporates the implications of each potential test result.

## Methods

### Development of the figure

The graphic presented here was developed as part of a project to develop guidance for writing plain language summaries for Cochrane diagnostic test accuracy (DTA) reviews. We have previously used similar graphics to present the results of work based on diagnosing urinary tract infection and myocardial infarction; however, these did not incorporate test consequences [7, 8].

We used a mixed methods approach consisting of focus groups, user testing, web-based surveys and a public engagement event to develop the test consequence graphic. Initial focus groups were conducted with a range of potential end-users including one with consumers (eight participants), one with journalists (nine participants) and one with clinicians (two participants). During the focus groups, we suggested a range of possible methods of presenting the numerical results of a systematic review including (1) text description based on sensitivity/specificity, (2) text description based on natural frequencies, (3) bulleted text description based on natural frequencies, (4) natural frequency graphic based on a hypothetical cohort of 1000 patients and (5) text-only description with no numbers (Additional file 1: Web Appendix A). The graphical display emerged as a clear preference across all three focus groups and was therefore adopted as the method that should be used to summarise the numerical results. Based on suggestions from the focus group, we restructured the graphic to flow from left to right rather than top to bottom and replaced the colours with different shaped boxes as there was concern that colours could be difficult to interpret for people who are colour blind. We also added additional boxes showing the consequences of each potential test result (true positive, false positive, true negative and false negative).

**Fig. 1:**
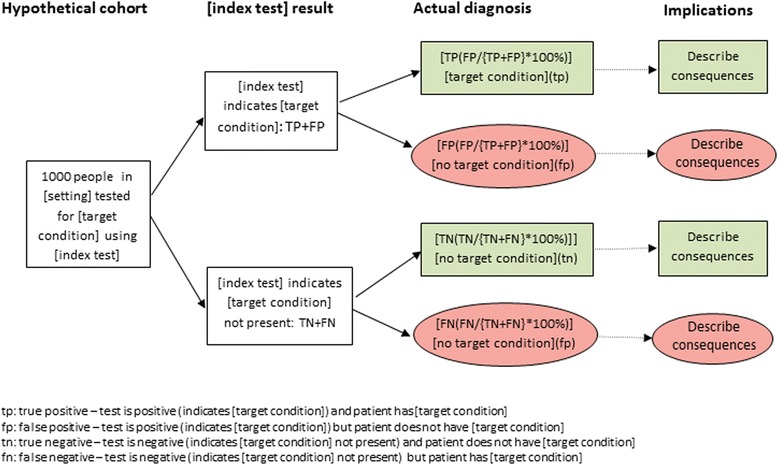
Template for development of example figure

The next stage of the development process involved one-on-one user testing with potential stake holders—four clinicians, one journalist, one commissioner, one Cochrane review author and one patient representative (Additional file 1: Web Appendix B). All supported the changes and no further changes were suggested. We then moved to a web-based survey to gain feedback from a wider group of participants. This was completed by 67 respondents including media representatives, methodologists, systematic review authors, health professionals and patient representatives. There was strong support for the use of a figure to summarise results and in particular the inclusion of information about consequences, although it was suggested that “implications” might be a more appropriate heading. Other changes made based on the survey included adding the colours back to the boxes—although the concerns regarding people with colour blindness were acknowledged, respondents felt that having the different shaped boxes would address these concerns and that colours in addition to this would be helpful for others. A refined version was produced that was considered as part of the second round of the survey (Additional file 1: Web Appendix C); no further changes were needed. The same version was used for a public engagement event where we presented the example plain language summary and figure to a group of 16- to 18-year-old school students. They suggested that the colours could be improved on the diagram by using less bright colours. They also felt that it was important to supplement the diagram with a text summary of results. This was added and has resulted in the final version (Fig. 1). This has been shared for further feedback during workshops at Cochrane Colloquia and with Cochrane DTA Editors. All have been positive about the diagram and no further changes have been suggested.

## Results

### Producing the figure

#### Structure

The diagram shows the consequences of using the index test (test under evaluation) in a hypothetical population of 1000 patients suspected of having the condition of interest (the target condition). Numerical information is presented in a natural frequency format. The size of the hypothetical cohort may be increased where the prevalence of the target condition is very low as research suggests that understanding is compromised when numbers being handled are very small [9, 10].

#### Data required

In order to populate the figure with natural frequencies, estimates of the accuracy of the index test (sensitivity and specificity) and prevalence of the target condition are needed. Sensitivity and specificity estimates may be derived from a single test accuracy study or from the meta-analysis of a DTA review. Where more than one estimate of sensitivity and specificity is available, the choice of which estimates to present will be influenced by factors such as precision and quality (the validity and applicability to the particular testing context being presented in the figure).

Estimating the prevalence of the target condition is a key first step in producing the graphic and will have implications for the applicability of the diagram. Estimates of the prevalence of the target condition can be derived from a number of sources. In a primary DTA study, an estimate of the prevalence of the target condition is obtained by dividing the number of individuals with the target condition by the total number tested. In a DTA review, the median prevalence, a pooled estimate of prevalence across all included studies or a prevalence estimate based on only higher quality (valid and applicable) studies, could be used. Other sources of prevalence data include disease registries, routine data sources, audits, epidemiological studies or professional opinion.

Table 1 illustrates how natural frequencies are derived from estimates of sensitivity, specificity and prevalence. Figure 1 shows how to use the information from Table 1 to construct the diagram.

**Table 1:** Calculating natural frequencies from estimates of sensitivity, specificity and prevalence

	Target condition present	Target condition absent	Total
Test positive	TP = *x* × sens	FP = *y* − (*y* × spec)	TP + FP
Test negative	FN = *x* − (*x* × sens)	TN = *y* × spec	FN + TN
	*x* = 1000 × *p*	*y* = 1000 − (1000 × *p*)	1000

*Sens* sensitivity, *spec* specificity, *p* prevalence, *TN* true negative—test is negative and patient does not have target condition, *FN* false negative—test is negative but patient has target condition, *TP* true positive—test is positive and patient has target condition, *FP* false positive—test is positive but patient does not have target condition, *x* number of patients with target condition, *y* number of patients without target condition

Our research to develop the diagram suggested that accompanying the diagram with a text description of results helps understanding. We suggest the following example of text that may be used:

“The results of this study/these studies indicate that in theory, if the [index test] were to be used in [setting] in a group of 1000 people where [*x*# (*x*%)] have [target condition] then:

- An estimated [TP + FP] will have an [index test] result indicating [target condition] and of these [FP(FP/{TP + FP} × 100%)] will not have [target condition]
- Of the [TN + FN] people with a result indicating that [target condition] is not present, [FN(FN/(TN + FN} × 100%)] will actually have [target condition]”

We illustrate the process of generating the graphic and accompanying textual explanation using the results of a DTA review of rapid tests for diagnosing group A streptococcus in children with sore throat [11]. This review reported a summary sensitivity of 85.7%, a summary specificity of 95.4% and the median prevalence of strep A infection across included studies was 30%.

Using Table 1 introduced above, we can obtain natural frequency estimates of the numbers of true positive (TP), false positive (FP), false negative (FN) and true negative (TN) test results that would be obtained if the rapid strep A test were applied to a hypothetical cohort of 1000 children in whom the prevalence of strep A infection was 30% (see Table 2).

**Table 2:** Natural frequency estimates based on the summary sensitivity and specificity and median prevalence of strep A infection from the systematic review of rapid tests for diagnosing group A streptococcus in children with sore throats

	Disease present	Disease absent	Total
Test positive	300 × 0.857 = 257	700 − (700 × 0.954) = 32	TP + FP = 289
Test negative	300 − (300 × 0.857) = 43	700 × 0.954 = 668	FN + TN = 711
	1000 × 0.3 = 300	1000 − 300 = 700	1000

**Fig. 2:**
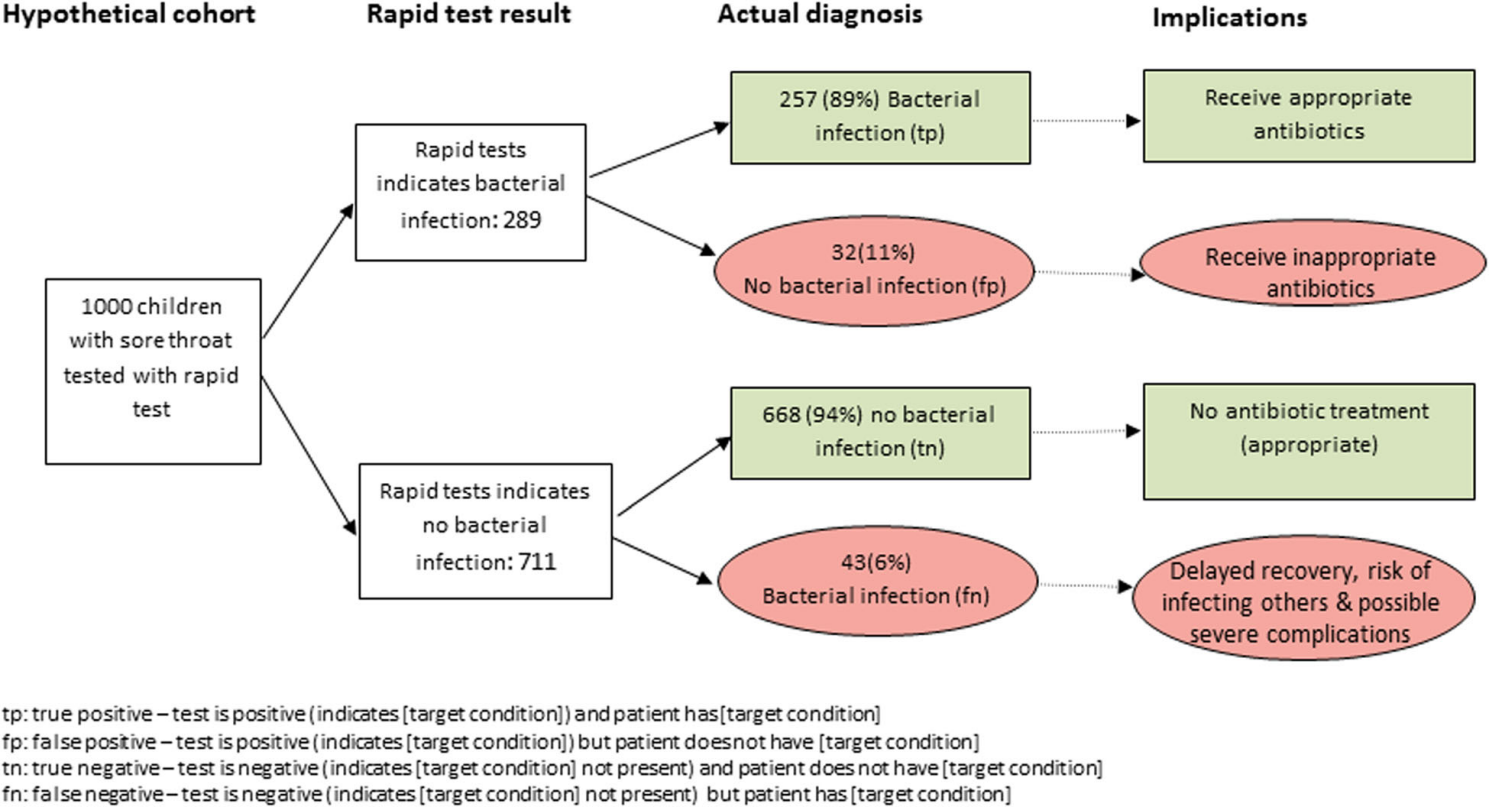
Figure showing results that would be obtained if a hypothetical cohort of 1000 children were tested for strep A infection using rapid tests

Figure 2 illustrates the graphic for the strep A example populated using natural frequencies from Table 2 and annotated with the potential downstream consequences of testing. The suggested accompanying text would read:

“The results of these studies indicate that in theory, if rapid tests were to be used in a group of 1000 children with sore throats, of which 300 (30%) are actually caused by bacterial infection then:

- An estimated 289 will have a rapid test result indicating that their sore throat is caused by a bacterial infection and of these 32 (11%) will not have a bacterial infection
- Of the 711 children with a rapid test result indicating that they do not have a bacterial infection, 43 (6%) will actually have one”

### Additional considerations

We suggest only including one flow diagram to summarise the main results to facilitate understanding and to draw attention to the most important findings from a piece of test accuracy research. Scenarios where multiple estimates of accuracy and/or prevalence may be present include:

- Presence of multiple estimates of accuracy for a single test (for example at different test positivity thresholds, in different patient groups, as a result of variation in quality of included studies in a systematic review)
- Presence of estimates of accuracy for more than one index test

A priori selection of primary analysis in test accuracy research may support the process of selection but are rarely sufficient to solve it completely. The most appropriate approach will vary across studies and will require clinical and methodological expertise.

The presence of variation in estimates of accuracy aside from that presented in the flow diagram should be highlighted in accompanying text, although the level of detail presented will be restricted by the need for plain language. Depending on the method of publication and dissemination, additional information may be made available as appendices or supplementary figures. Availability online offers the potential to make the figure interactive so that different estimates of prevalence, sensitivity and specificity can be selected with the numbers included in the diagram automatically updating.

## Discussion

We consider that the main strength of our proposed figure is that it will help researchers communicate the results of diagnostic research in a simple, easy to access format and encourage meaningful application of research findings to practice. Key to this is linking estimates of test accuracy to potential downstream consequences of testing. In addition, greater consistency in the use of terminology and presentation format should assist with the familiarisation of test accuracy research.

A limitation is the need to select single estimates of prevalence, sensitivity and specificity. Natural frequency representation of patient outcomes of testing in the figure will only apply for the estimate of prevalence, sensitivity and specificity used in the figure. If the prevalence given in the figure is different to the estimate of prevalence that a reader is interested in, then the numbers will not be applicable. It is also not possible to convey uncertainty around estimates of accuracy in the figure. It is therefore important to stress the setting specific nature of test accuracy estimates to users of the graphic. One potential method of overcoming these limitations would be to make an interactive version of the figure available online so that different estimates of prevalence, sensitivity and specificity can be selected with the numbers included in the diagram automatically updating.

## Conclusions

The test consequence graphic provides a tool to help researchers communicate the results of diagnostic research in a simple, easy to access format and encourage meaningful application of research findings to practice. Linking estimates of test accuracy to potential downstream consequences of testing is a key feature of the proposed graphic.

## Additional file

Additional file 1: Web Appendix: Evolution of graphical display. (DOCX 106 kb)

## Abbreviations

DTA: Diagnostic test accuracy (DTA)
FN: False negative
FP: False positive
TN: True negative
TP: True positive
