# Supplementary material for: Understanding test accuracy research: a test consequence graphic
Source: Diagn Progn Res. 2018 Feb 1;2:2. doi: 10.1186/s41512-017-0023-0 (PMC6460548; doi:10.1186/s41512-017-0023-0)
Supplement: Supplementary file 1 — Web Appendix: Evolution of graphical display. (DOCX 106 kb) [file 41512_2017_23_MOESM1_ESM.docx]

# Web Appendix: Evolution of Graphical Display

## A. Methods of presenting results considered in focus groups

### Option 1: Sensitivity and Specificity

The IQCODE had a sensitivity of 95%, this means that it most people with dementia would be identified by the IQCODE. It had a specificity of 89% meaning that 11% of those without dementia would also have a positive result. The IQCODE performed less well in specialist memory clinics and psychiatry wards; authors reported more false positive and false negative results in these settings.

### Option 2: Natural frequencies embedded in text

Out of 1000 people tested using the IQCODE, we would expect 612 to have a low score (less than 3.3) of which only 13 would have dementia (false negative results). It is probably less helpful for ‘ruling in’ a diagnosis of dementia. We would expect 388 people to have a high IQCODE score (above 3.3) and of these 141 would not have dementia (false positive results).

### Option 3: Natural frequencies embedded in bulleted text

Using the IQCODE for diagnosing dementia in the general hospital setting:

- Of every 1000 people tested using the IQCODE, an estimated 612 will have a negative result and of these 13 will actually have dementia
- Of the 388 people with a positive IQCODE result, 141 will be incorrectly classified as having dementia

### Option 4: Graphical display incorporating natural frequencies

The figure below shows the expected results in a hypothetical group of a 1000 patients tested for dementia using the IQCODE:

1000 people tested for dementia

612 test negative

388 test positive

141

no dementia

247

dementia

13

dementia

599

no dementia

### Option 5: Text only summary

The IQCODE can help in 'ruling out' dementia in the general hospital setting. This means that if a person has a low IQCODE score, they are unlikely to have dementia. Yet if a person has a high score, that person does not necessarily have dementia.

## B. Figure used for User Testing and Web Based Survey Round 1

The figure below shows the expected results in a group of 1000 patients tested for dementia using the IQCODE:

| **Hypothetical cohort** | **IQCODE result** | **Actual diagnosis** | **Consequence** |
| --- | --- | --- | --- |

445

no dementia (tn)

No intervention, consider other diagnoses

IQCODE indicates dementia not present: 470

1000 people tested for dementia using the IQCODE

Appropriate intervention

475

Dementia (tp)

IQCODE indicates dementia: 530

tn: true negative – test is negative (indicates dementia not present) and patient does not have dementia; fn – test negative but patient has dementia; tp: true positive – test positive (indicates dementia) and patient has dementia; fp: false positive – test is positive but patient does not have dementia

## C. Figure used for Web-Based Survey Round 2 and Public Engagement Event

The review indicates that if the IQCODE were to be used in a group of 1000 people in mixed hospital settings (general hospital and specialist memory clinics) where 500 (50%) have dementia, the following results would be observed:

|  | **IQCODE result** | **Actual diagnosis** | **Implications** |
| --- | --- | --- | --- |

475 (48%)

Dementia (tp)

IQCODE indicates dementia: 530

Appropriate intervention

1000 people in mixed hospital settings tested for dementia using the IQCODE

No intervention, consider other diagnoses

445 (45%)

no dementia (tn)

IQCODE indicates dementia not present: 470

tn: true negative – test is negative (indicates dementia not present) and patient does not have dementia;

fn – test negative but patient has dementia;

tp: true positive – test positive (indicates dementia) and patient has dementia;

fp: false positive – test is positive but patient does not have dementia

## D. Final Figure

The review included 13 relevant studies with a total of 2745 participants. The results of these studies indicate that in theory, if the IQCODE were to be used in mixed hospital settings) in a group of 1000 people where 500 (50%) have dementia:

- An estimated 625 will have an IQCODE result indicating dementia and of these 170 (27%) will not have dementia
- Of the 375 people with a result indicating that dementia is not present, 45 (12%) will actually have dementia

|  | **IQCODE result** | **Actual diagnosis** | **Implications** |
| --- | --- | --- | --- |

455 (73%)

Dementia (tp)

Specialist assessment, appropriate intervention

IQCODE indicates dementia: 625

1000 people in mixed hospital settings tested for dementia using the IQCODE

No intervention, consider other diagnoses

330 (88%)

no dementia (tn)

IQCODE indicates dementia not present: 375

tn: true negative – test is negative (indicates dementia not present) and patient does not have dementia;

fn: false negative – test is negative (indicates dementia not present) but patient has dementia;

tp: true positive – test is positive (indicates dementia) and patient has dementia;

fp: false positive – test is positive (indicates dementia) but patient does not have dementia
